# Intimate Partner Violence is Associated with Increased CD4^+^ T-Cell Activation Among HIV-Negative High-Risk Women

**DOI:** 10.20411/pai.v1i1.120

**Published:** 2016-09-15

**Authors:** Ameeta S. Kalokhe, Chris C. Ibegbu, Surinder P. Kaur, Rama R. Amara, Mary E. Kelley, Carlos del Rio, Rob Stephenson

**Affiliations:** 1 Emory School of Medicine, Division of Infectious Diseases, Atlanta, Georgia; 2 Emory Rollins School of Public Health, Department of Global Health, Atlanta, Georgia; 3 Yerkes National Primate Research Center, Emory University, Atlanta, Georgia; 4 Emory Vaccine Center, Department of Microbiology and Immunology, Atlanta, Georgia; 5 Emory Rollins School of Public Health, Department of Biostatistics and Bioinformatics, Atlanta, Georgia; 6 University of Michigan School of Nursing, Department of Health Behavior and Biological Sciences, Ann Arbor, Michigan

**Keywords:** intimate partner violence, gender-based violence, spouse abuse, CD4-positive T-lymphocytes, lymphocyte activation, HIV infections, risk, humans, regulatory T-lymphocytes

## Abstract

**Background:**

Biological pathways mediating the link between intimate partner violence (IPV) and increased HIV risk remain unexplored. We hypothesized that IPV-induced stress negatively affects HIV systemic immune defenses and aimed to evaluate whether IPV was associated with immune profiles linked to HIV susceptibility: CD4 activation and diminished regulatory T-cell (Treg) frequency.

**Methods:**

Seventy-five HIV-negative high-risk women were surveyed regarding their IPV experience. They provided blood, urine, and (if present) genital ulcer samples for cortisol, immune assays, and STI testing. Using flow cytometry, we assessed activated CD4^+^ T-cell (%HLA-DR^+^/ CD38^+^) and Treg (%CD4^+^CD25^+^FoxP3^+^) frequencies and phenotyping. Nonparametric tests evaluated the association between IPV and immune outcomes. Multivariate regression explored confounding and moderation of the IPV-CD4 activation pathway.

**Results:**

Lifetime IPV was associated with increased CD4^+^ activation (r = 0.331, *P* = 0.004), a shift in CD4^+^ phenotype from naïve to effector memory (r = 0.343, *P* = 0.003), and a decrease in naive (%HLA-DR^+^/CD45RA^-^) Treg frequency (r = -0.337, *P* = 0.003). Experiencing IPV over the past year had similar trends. After controlling for sexual IPV, lifetime physical and psychological abuse remained significantly associated with CD4^+^ activation (*P* = 0.004 and *P* = 0.033, respectively). After controlling for race (the only covariate linked to activation), the lifetime IPV-CD4 activation association remained significant (*P* = 0.012). Alcohol use and depression were identified as potential pathway moderators.

**Conclusion:**

Our data is the first to suggest an immune link between IPV and HIV, and may help explain differences at the individual level in HIV susceptibility and response to biological HIV prevention strategies. The association of psychological and physical abuse with CD4 activation independent of sexual abuse further supports the existence of a stress-induced immune pathway.

**STANDFIRST**

Recent and longitudinal intimate partner violence experience among HIV high-risk women is associated with increased CD4^+^ T-cell activation and a shift in CD4^+^ T-cell and Treg phenotype.

## INTRODUCTION

Intimate partner violence (IPV) is defined as physical violence, sexual violence, stalking, and psychological aggression by a current or former intimate partner [1]. Nationally, 35% of heterosexual women report experience of lifetime physical and sexual abuse and 48% report lifetime psychological abuse [2, 3]. Several studies have linked IPV experience to increased risk of HIV infection [4–12] and other negative physical and mental health outcomes [13–18]. To date, the association between IPV and HIV has largely been explored through a behavioral lens with many studies implicating increased substance abuse [19], inconsistent condom use [19], engagement in higher risk sexual activities like transactional [7] and unprotected anal sex [20], and having multiple and high-risk sexual partners [19, 21]. It is of interest that after controlling for high-risk behaviors, the link between IPV and HIV risk remains [6–8, 10, 22, 23], indicating the association between IPV and HIV cannot be explained solely through behavioral mechanisms.

Biological pathways between IPV and HIV remain relatively unexplored. Two potential biological pathways explaining the increased HIV susceptibility include mucosal-level changes due to sexual violence-induced trauma and stress-induced systemic immune changes. Recent literature demonstrates that in addition to sexual IPV increasing HIV risk, experience of physical and psychological IPV alone increases susceptibility, and experiencing multiple forms of IPV (i.e. physical, psychological, and sexual) may have an additive effect on HIV risk [4, 7, 8, 24]. Thus, while sexual IPV may cause potential changes at the mucosal level, this form of IPV together with the other IPV forms, with which it often co-occurs, may independently and additively lead to stress-induced changes in the systemic immune defenses against HIV.

Evidence that IPV experience may impact systemic immunity comes from various sources. First, IPV has been linked to an increased risk of communicable diseases, including sexually transmitted infections (STIs) [25], urinary tract infections [26], and respiratory tract infections [27]. Second, although minimally explored, IPV has been associated with poor humoral responses and T-cell mitogen responses. A study comparing salivary samples of women who suffered physical or psychological IPV with those of non-abused controls demonstrated that the abused women had lower herpes simplex virus (HSV)-1 virus neutralization capacity and HSV-1 specific antibody (HSV-sIgA) production [28]. A follow-up study performed 3 years later demonstrated that abuse cessation was associated with significant improvement in HSV-1 neutralization capacity and recovery of HSV-sIgA levels [29]. Additionally, preliminary studies comparing phytohemagglutinin-stimulated cellular responses among women who report IPV with women who do not, suggest IPV may negatively affect T-cell mitogen responses [30, 31]. Third, in antiretroviral-naïve HIV-infected individuals, psychological IPV has been associated with a more rapid CD4^+^ T-cell decline [32].

In this study, we hypothesized that the increased HIV susceptibility that IPV survivors incur is due to the impact of stress from the IPV experience on CD4^+^ T-cell activation and the frequency of circulating regulatory T cells (Tregs). Studies exploring immune profiles of individuals with HIV high-risk exposures but who remain HIV-negative (“HIV resistant”), have consistently linked increased CD4^+^ T-cell activation to heightened HIV susceptibility [33–37]. For HIV to disseminate and seroconversion to occur, infection of activated CD4^+^ T-cells is critical, as activated CD4^+^ T-cells more efficiently produce HIV virions and transmit infection to more distant cells than do resting CD4^+^ T-cells [38, 39]. Tregs have been hypothesized to contribute to HIV natural immunity through suppressing CD4^+^ T-cell activation and directly inhibiting HIV replication. HIV-resistant individuals have higher frequencies of Tregs [36]. Similarly, HIV vertically-exposed, uninfected infants demonstrate high frequencies of Tregs and low levels of CD4^+^ T cell activation [40]. Lastly, individuals with increased CD4^+^ T-cell activation and lower frequencies of Tregs have increased susceptibility to *in vitro* HIV infection [41].

**Figure 1.**
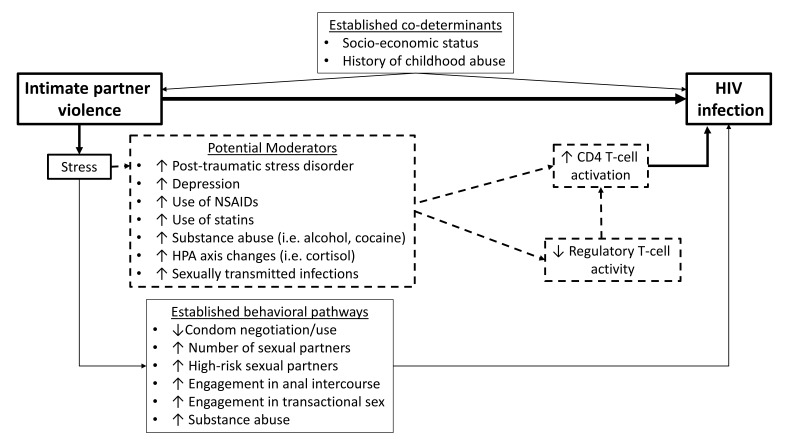
**Behavioral and biological pathways linking intimate partner violence (IPV) experience and HIV susceptibility.** Established behavioral pathways linking IPV experience and HIV susceptibility (closed boxes). Potential biological pathways between IPV experience and HIV susceptibility being tested in the described studies (perforated boxes).

Thus, in a sample of HIV-negative high-risk women, we sought to explore a systemic immune link between experiencing IPV and HIV susceptibility (Figure 1). In this paper, we present preliminary evidence of an association between recent and long-term physical, sexual, and psychological IPV experience and 1) the level of CD4^+^ T-cell activation, 2) a shift in CD4^+^ T-cell pheno-type from naïve to central and effector memory, and 3) a shift in Treg phenotype from naïve to terminal effector. This preliminary evidence has the potential to increase our current limited understanding of the biological pathways between IPV experience and the risk of HIV acquisition.

## METHODS

### Experimental design

The study used a cross-sectional design. Between March and December 2014, 85 HIV-negative high-risk women aged 18–50 years were enrolled from an urban infectious disease clinic in Atlanta, Georgia. All interviews were computer-assisted, conducted one-on-one in a private clinic room, and of 40–60 minutes duration. After provision of written informed consent, participants completed a screening questionnaire, and underwent rapid oral HIV testing, and urine pregnancy testing to assess study eligibility (described below). Those who met study inclusion criteria completed the follow-up questionnaire and underwent a blood draw into heparinized cell preparation tubes (CPT, BD Biosciences, Franklin, New Jersey). They also underwent an external pelvic exam to evaluate for active genital ulcers with a swab of open ulcer(s) for HSV culture if present. In accordance with the World Health Organization guidelines [42], all participants were given contact information of community IPV support services and offered referral. The study was reviewed and approved by the Emory University Institutional Review Board and the Grady Research Oversight Committee and the approved protocol was followed in the conduct of the described research.

### Subjects

Subjects were deemed eligible if they were a HIV-negative “high-risk” woman aged 18–50 years. In accordance with the Women's Interagency HIV Study (WIHS) criteria, a woman was defined as “HIV high-risk” if she self-reported one of the following in the prior five years: 1) use of intravenous drugs, crack, cocaine, or methamphetamines, 2) an STI, 3) unprotected vaginal or anal sexual intercourse with ≥ 3 men, 4) vaginal or anal sexual intercourse with > 6 men, 5) sex with a known HIV-positive man, 6) transactional sex, or 7) a sexual partner who used the aforementioned drugs, engaged in transactional sex, had an STI, sexual intercourse with men, or sex with an HIV-positive partner. Subjects were excluded if they had a positive pregnancy test or positive rapid HIV test, reported having chronic hepatitis B or C, an autoimmune disease, or immunosuppressive condition, or using immunosuppressive medications (including recent systemic steroids). Convenience sampling was used to recruit subjects from the Greater Atlanta community (i.e. shopping malls, universities, crack houses, public health fairs, and churches). Additionally, some participants were referred from the Atlanta WIHS HIV-negative cohort, a clinical drug trial, and from databases of prior HIV-prevention trials.

### Survey Instruments

Lifetime and past-year psychological abuse were assessed using the Index of Psychological Abuse (IPA) (Cronbach alpha=0.97), while lifetime and past-year physical and sexual abuse were assessed using the Severity of Violence Against Women Scale (SVAWS) (Cronbach alpha=0.95) [43]. To explore potential confounding, information about demographics (i.e. age, income, education, race, sexual orientation, employment, relationship status, number of children, and homelessness), experience of child abuse, and discrimination (using the Everyday Discrimination Scale [44]) was collected. To assess for potential effect moderation, participants were questioned about post-traumatic stress disorder (using the PTSD Symptom Scale [45]), depression (using the CESD-10 [46]), and the use of nonsteroidal anti-inflammatory drugs (NSAID), alcohol and cocaine during the past week.

### Clinical Laboratory Tests

The OraQuick ADVANCE Rapid HIV-1/2 Antibody Test (OraSure Technologies, Inc., Bethlehem, Pennsylvania) and Fisher HealthCare^TM^ Sure-Vue^TM^ Urine hCG tests (Fischer Scientific, Waltham, Massachusetts) were used to assess HIV status and pregnancy. To evaluate for potential moderation by STIs, HSV culture of genital ulcer specimens, serum RPR for syphilis, and urine Chlamydia trachomatis/Neisseria gonorrhoeae RNA-TMA were performed off-site by Quest Diagnostics (Atlanta, Georgia). To test for moderation by cortisol, blood samples were collected between 8 am–10 am and plasma cortisol was measured using the Cortisol LCMS (Emory University Biomarker Core, Atlanta, Georgia). The remaining whole blood was used for immune assays described below.

### Blood Processing and PMBC Isolation

First, whole blood was used for immunophenotyping. The remaining sample was used to isolate and cryopreserve peripheral blood mononuclear cells (PBMC) in 90% Fetal Bovine Serum (FBS, Hyclone, South Logan, UT) and 10% dimethyl sulphoxide (DMSO, Sigma-Aldrich, St. Louis, MO) as described previously [47].

### Immunophenotyping

A whole blood flow cytometry technique was employed as previously described [47]. Whole-blood samples (200ul) were stained at room temperature for 20 minutes. The following directly conjugated monoclonal antibodies (mAbs) were used: anti-CD3 (clone UCHT1, BD Biosciences), anti-CD4 (clone L-200, BD Biosciences), anti-CD8 (clone SK1, BD Biosciences), anti-CD27 (clone L128, BD Biosciences), anti-CD38 (clone LS-198, Beckman Coulter), anti-CD45RA (clone AbB11, Beckman Coulter) and anti-HLA-DR (clone L243, Biolegend). Red cells were lysed and fixed in FACS lysing solution (BD Biosciences) for 10 minutes in the dark, washed twice in FACS buffer (phosphate-buffered saline (PBS) containing 2% bovine serum albumin and 0.1% NaN_3_), and fixed with 1% formalin in PBS. Cells were acquired on an LSR-II flow cytometer (BD Biosciences).

### Staining for Foxp3+ Cells

Cryopreserved PBMC were thawed and rested for 6 hours at 37°C, 5% CO_2_ in RPMI 1640 medium (Lonza) containing 10% FBS, 2mM glutamine, 100 IU/ml penicillin, and 100 μm/ml streptomycin. 1x10^6^ PBMC were stained with a marker to distinguish live and dead cells (Invitrogen) followed by surface staining with the following directly conjugated mAbs: anti-CD3, anti-CD4, anti-CD25 (clone 4E3, Miltenyi Biotech), anti-CD45RA, and anti-HLA-DR for 25 minutes at room temperature. Cells were then washed once with FACS wash buffer and fixed with 1X fix/ perm buffer (Tonbo Biosciences) for 60 minutes at room temperature, then washed again with 1X perm buffer solution and incubated in this buffer solution for 10 minutes, washed again with 1X perm buffer solution, and incubated for 45 minutes with mAb to Foxp3 (clone 206D, Biolegend). An isotype-matched antibody was used as a control (Biolegend). Cells were then washed with 1X perm buffer solution, followed by an additional wash with FACS wash buffer, re-suspended in 1% formalin in PBS, and acquired by the LSR-II flow cytometer.

### Statistical Analysis

**Supplemental Figure 1.**
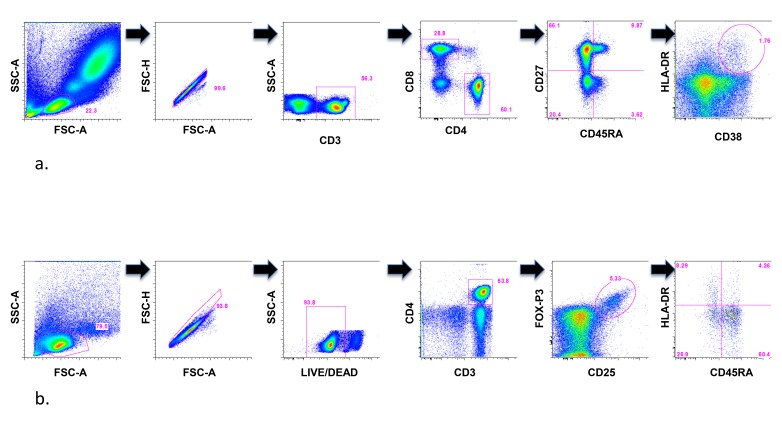
Applied gating strategy.

Behavioral data was collected using Survey Gizmo and transferred into Excel. SK and CCI conducted the analysis of the raw flow data in FlowJo software version 9.2 (Tree Star Inc.) and were blinded to the behavioral and clinical data. (Supplementary Figure 1 depicts gating strategy). The analyzed flow data was transferred to Excel, where it was consolidated with the behavioral dataset, STI, and cortisol results. The entire dataset was exported to SPSS for further analysis and Graph-Pad Prism 6.0 for graphing. The distributions of the IPV variables, the covariates, and immune outcomes were assessed to determine analysis strategy. Because sexual abuse was reported with low frequency and varies in its extent, it was dichotomized for further analysis.

Nonparametric tests (i.e. Mann-Whitney tests and nonparametric correlations) were used to explore the association between IPV, covariates, and the immune outcomes. Multivariable regression was used to 1) assess whether experience of physical and psychological abuse were associated with CD4 activation independent of sexual abuse, 2) explore possible confounding of the IPVCD4 relationship, and 3) assess whether there was moderation by cortisol, PTSD, depression, child abuse, and use of NSAIDs, cocaine, or alcohol. Although statin and STI data was originally collected to evaluate for moderation of the IPV-activation pathway, low frequency of affirmative responses rendered evaluation unnecessary and not possible in this study sample. A two-tailed *P* value of ≤ 0.05 was deemed statistically significant. For the moderator analyses, indicator (yes/no) variables were calculated for the following variables: PTSD diagnosis was defined using the PSS data as in Foa *et al* [45], depression was defined as CES-D10 total ≥ 10, and alcohol use as consuming more than 7 drinks per week.

### Role of the Funding Source

Funding was provided by NIH/NIAID through Emory CFAR (P30 AI050409). The funding body played no role in study design or conduct, data analysis or interpretation, or decisions regarding manuscript publication.

## RESULTS

### Study Participants

Of 85 women who provided informed consent, and underwent screening for the study, 75 were eligible and completed all study visit requirements (Table 1). Four women were excluded for not meeting HIV risk criteria, 2 for being HIV-infected, 2 for being hepatitis C infected, and 2 because we were unable to obtain an adequate blood sample. The average age was 34 +/- 8 years and the majority (79% or 59/75) were Black/African American and (75% or 56/75) had an annual income of less than $10,000. Additional demographic, socio-behavioral, and clinical measurements are listed in Table 1.The average past-year score for the IPA was 37.04 +/- 24.72, for the physical violence SVAWS subscale 29.92 +/- 25.97, and for the sexual violence SVAWS subscale 3.55 +/- 4.73. The average lifetime score for the IPA was 56.47 +/- 33.52, for the physical violence SVAWS subscale 48.95 +/- 36.17, and for the sexual violence SVAWS subscale 5.85 +/- 6.48.

**Table 1.** **Associations with CD4 activation (outcome) and demographic, behavioral, and clinical measures (predictors) in n = 75 subjects**. SD = standard deviation; EDS = The Everyday Discrimination; PTSD = post-traumatic stress disorder; PSS = PTSD Symptom Scale; CES-D10 = 10-item Center for Epi-demiologic Studies of Depression Short Form; IPA = Index of Psychological Abuse; SVAWS=Severity of Violence Against Women Scale; NSAID=nonsteroidal anti-inflammatory drug; STI = sexually transmitted disease; ^‖^Income was measured as a 4-level variable where income < $10,000=1, $10,000 - 20,000 = 2, $20,000 - 40,000 = 3, and > $40,000 = 4.

Variable	Mean (SD)	Rho			P
Age, (years)	34.1 (8.5)	0.181			0.121
Income^‖^	1.4 (0.7)	0.025			0.832
Discrimination (EDS score)	17.6 (7.1)	−0.187			0.109
PTSD (PSS score)	23.5 (13.8)	0.247			**0.034**
Depression (CES-D10 score)	13.6 (6.8)	0.125			0.286
Psychological abuse (IPA score)					
Lifetime	19.0 (9.5)	0.326			**0.004**
Past-year	36.6 (24.2)	0.261			**0.024**
Physical abuse (SVAWS subscore)					
Lifetime	18.6 (11.3)	0.312			**0.006**
Past-year	28.6 (25.2)	0.287			**0.013**
Sexual abuse (SVAWS subscore)					
Lifetime	2.2 (2.1)	0.228			**0.049**
Past-year	3.3 (4.6)	0.113			0.333
Cortisol	8.1 (4.9)	0.025			0.835
					

Variable	N (%Positive)	Median		Z*	P
		No	Yes		
Education ≤ high school diploma	41 (48.2)	0.598	0.593	−0.569	0.569
Race, non-white	71 (83.5)	0.457	0.615	−2.053	**0.040**
Employed	29 (34.1)	0.587	0.621	−0.087	0.931
Presently in an intimate relationship	37 (43.5)	0.603	0.596	−0.102	0.919
Has children	59 (69.4)	0.576	0.598	−1.141	0.254
Current homelessness	8 (9.4)	0.590	0.759	−1.236	0.217
Sexual orientation, non-heterosexual	28 (32.9)	0.590	0.617	−0.714	0.475
History of abuse as child	58 (75.3)	0.536	0.598	−1.309	0.191
Cocaine, prior 3 months	20 (26.0)	0.590	0.711	−1.535	0.125
Alcohol, past week	25 (32.5)	0.589	0.620	−1.295	0.195
NSAID use	43 (55.8)	0.590	0.597	−0.854	0.393
Statin use	1 (1.3)	***	***	***	***
STI	1 (1.3)	***	***	***	***

### Experience of IPV is Associated with Increased CD4 Activation

**Figure 2.**
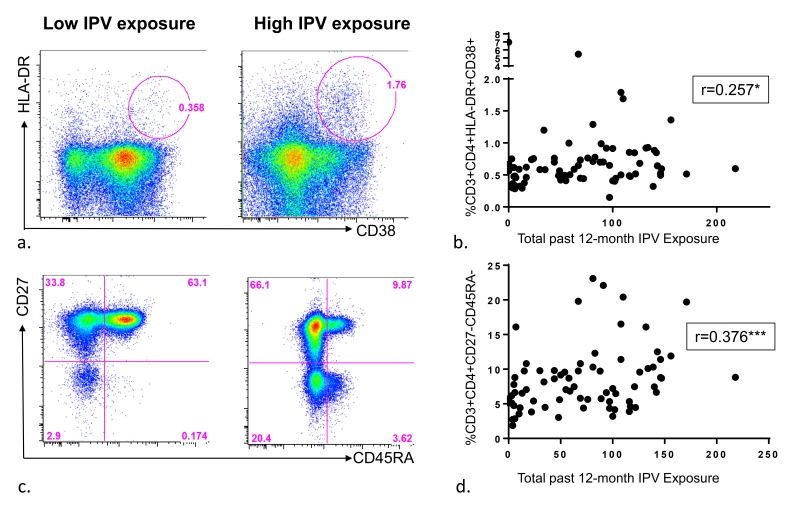
**Association between past-year intimate partner violence (IPV) experience and CD4^+^ T-cell activation and memory phenotype among HIV high-risk women. (A)** Representative flow diagrams demonstrating CD4^+^ T-cell activation (%HLA-DR^+^/CD38^+^) in the whole blood of two donors with low versus high reporting of past-year IPV experience. **(B)** Summary data from 75 HIV high-risk women demonstrating past-year IPV reporting is significantly associated with CD4^+^ T-cell activation (%HLADR^+^/CD38^+^) in whole blood. **(C)** Representative flow diagrams depicting frequencies of CD4^+^ T-cell memory phenotypes using CD27 and CD45RA markers in the whole blood of two donors with low versus high reporting of past-year IPV experience, and **(D)** Summary data from 75 HIV high-risk women demonstrating past-year IPV reporting is significantly associated with an increase in CD4^+^ effector memory T-cell population (%CD27^-^CD45RA^-^).

**Figure 3.**
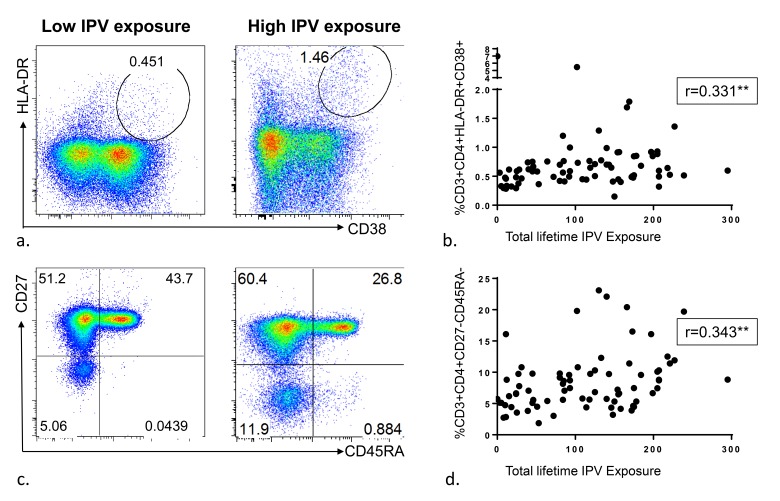
**Association between lifetime intimate partner violence (IPV) experience and CD4^+^ T-cell activation and memory phenotype among HIV high-risk women. (A)** Representative flow diagrams demonstrating CD4^+^ T-cell activation (%HLA-DR^+^/CD38^+^) in the whole blood of two donors with low versus high reporting of lifetime IPV experience. **(B)** Summary data from 75 HIV high-risk women demonstrating lifetime IPV reporting is significantly associated with CD4^+^ T-cell activation (%HLA-DR^+^/ CD38^+^) in whole blood. **(C)** Representative flow diagrams depicting frequencies of CD4^+^ T-cell memory phenotypes using CD27 and CD45RA markers in the whole blood of two donors with low versus high reporting of lifetime IPV experience, and **(D)** Summary data from 75 HIV high-risk women demonstrating lifetime IPV reporting is significantly associated with an increase in CD4^+^ effector memory T-cell population (%CD27^-^CD45RA^-^).

To assess whether experience of IPV was associated with higher CD4^+^ T cell activation, participants were surveyed regarding past-year and lifetime physical, sexual, and psychological IPV, and whole blood samples were stained using anti-CD38 and anti-HLA-DR and acquired by flow cytometry (Figure 2A, 2B, 3A, 3B). Both recent (during the past year) and longitudinal (lifetime) IPV experience was associated with higher frequencies of activated HLA-DR^+^/CD38^+^ CD4^+^ T-cells (r = 0.257, *P* = 0.026 and r = 0.331, *P* = 0.004 respectively). Furthermore, all forms of IPV were associated with increased CD4^+^ T-cell activation [Table 1]. Of note, neither past-year nor lifetime IPV experience was associated with CD8^+^ T-cell activation (data not shown).

Next, because the association between IPV and systemic CD4 activation could be explained by stress-induced changes or through sexual abuse-related vaginal mucosal trauma, we sought to explore whether the IPV-CD4 activation link persisted after controlling for sexual abuse. Interestingly, both psychological and physical abuse remained significantly associated with CD4 activation after controlling for sexual abuse (*P* = 0.033 and *P* = 0.004, respectively).

### Experience of IPV is Associated with a Shift in CD4 Phenotype

To explore whether experience of IPV was associated with a shift in CD4 T-cell phenotype, participants' whole blood samples were stained using anti-CD45RA and anti-CD27 and analyzed by flow cytometry. Higher total scores of past-year IPV were associated with lower frequencies of naïve CD4 T-cells (%CD45RA^+^CD27^+^ CD4^+^ T cells) (r= -0.345, *P*=0.002) and higher frequencies of central memory (%CD45RA^-^CD27^+^CD4^+^) (r=0.213, *P*=0.07) and effector memory CD4^+^ T cells (%CD45RA^-^CD27^-^ CD4^+^), (r= 0.376, *P* = 0.001) (Figure 2C, 2D). Analysis of lifetime IPV experience yielded similar results, with significant phenotypic shift in CD4^+^ T cells from naïve (r = -0.344, *P* = 0.003) to central memory (r = 0.227, *P* = 0.05) and effector memory (r = 0.343, *P* = 0.003) (Figure 3C, 3D). This significant trend was seen across all forms of IPV (physical, sexual, and psychological) when analyzed separately as well.

### Experience of IPV is Associated with a Shift in Phenotype of Regulatory T Cells

**Figure 4.**
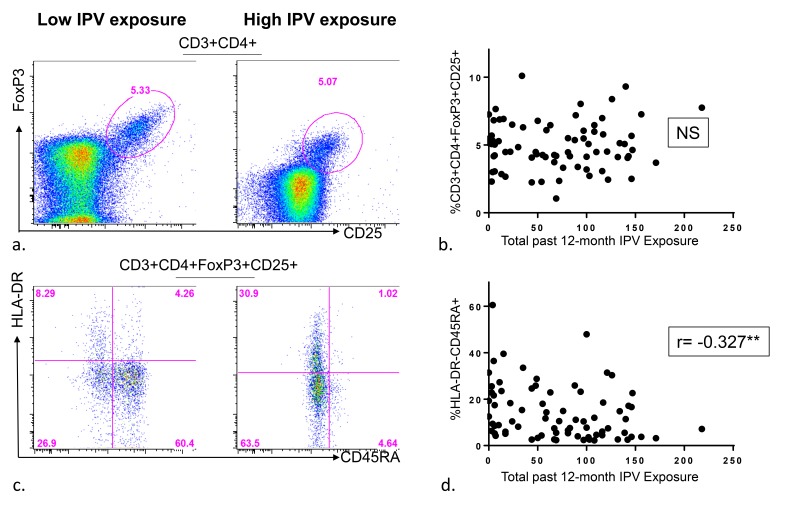
**Association between past-year intimate partner violence (IPV) experience and regulatory (CD4^+^Foxp3^+^CD25^+^) T-cell frequency and phenotype among HIV high-risk women. (A)** Representative flow diagrams demonstrating frequencies of regulatory (CD4^+^Foxp3^+^CD25^+^) T-cells in the PBMC of two donors with low versus high reporting of past-year IPV experience. **(B)** Summary data from 75 HIV high-risk women demonstrating past-year IPV experience was not significantly associated with frequency of regulatory (CD4^+^FoxP3^+^CD25^+^) T-cells in PBMC. **(C)** Representative flow diagrams depicting frequencies of regulatory (CD4^+^FoxP3^+^CD25^+^) T-cell phenotypes using CD45RA and HLA-DR markers in the PBMC of two donors with low versus high reporting of past-year IPV experience. **(D)** Summary data from 75 HIV high-risk women demonstrating past-year IPV experience is negatively associated with the frequency of naïve regulatory T-cells (HLA-DR^-^CD45RA^+^CD4^+^FoxP3^+^CD25^+^) in PBMC.

**Figure 5.**
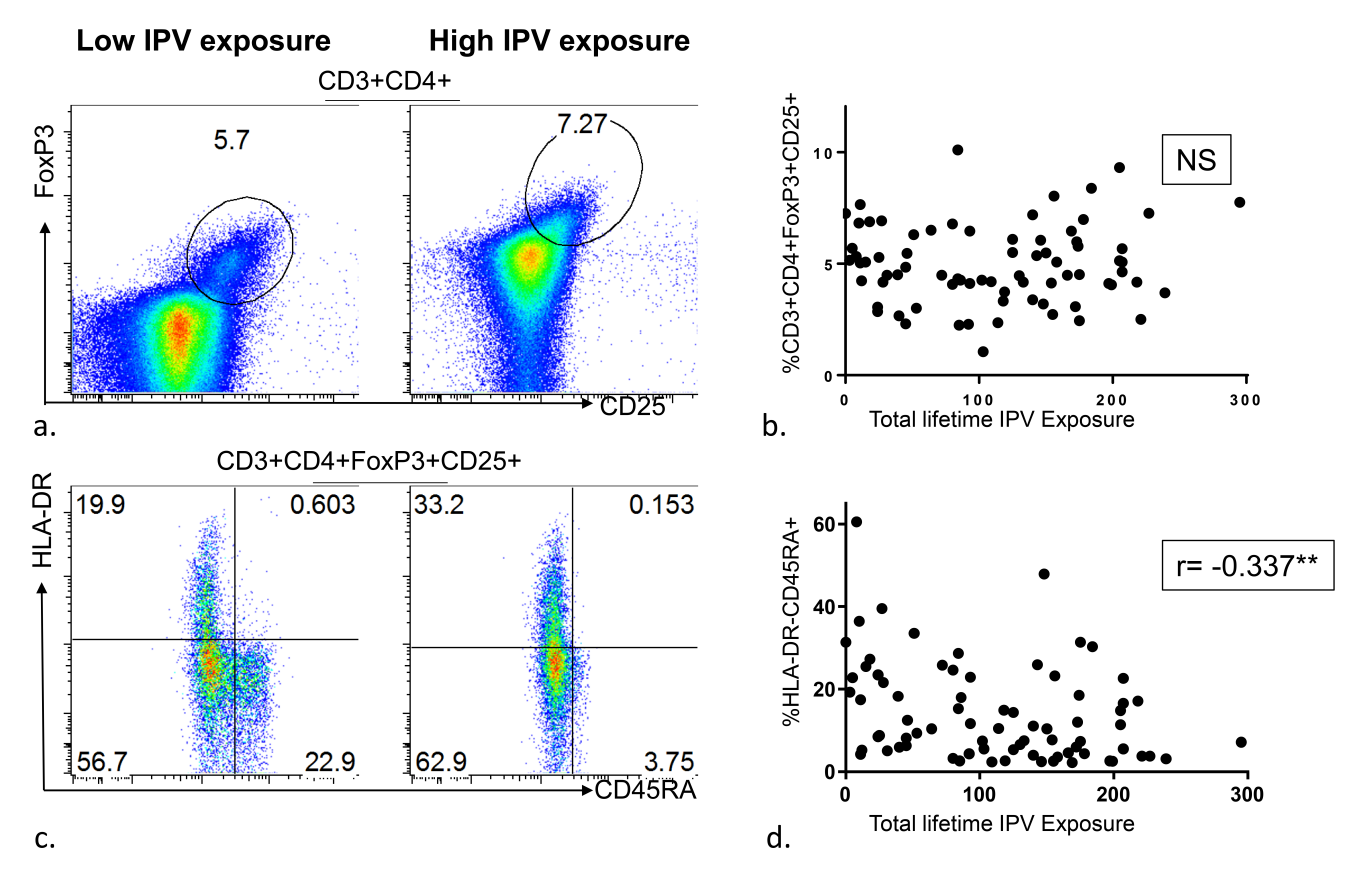
**Association between lifetime intimate partner violence (IPV) experience and regulatory (CD4^+^Foxp3^+^CD25^+^) T-cell frequency and phenotype among HIV high-risk women. (A)** Representative flow diagrams demonstrating frequencies of regulatory (CD4^+^Foxp3^+^CD25^+^) T-cells in the PBMC of two donors with low versus high reporting of lifetime IPV experience. **(B)** Summary data from 75 HIV high-risk women demonstrating lifetime IPV experience was not significantly associated with frequency of regulatory (CD4^+^FoxP3^+^CD25^+^) T-cells in PBMC. **(C)** Representative flow diagrams depicting frequencies of regulatory (CD4^+^FoxP3^+^CD25^+^) T-cell phenotypes using CD45RA and HLA-DR markers in the PBMC of two donors with low versus high reporting of lifetime IPV experience. **(D)** Summary data from 75 HIV high-risk women demonstrating lifetime IPV experience is negatively associated with the frequency of naïve regulatory T-cells (HLA-DR^-^CD45RA^+^CD4^+^FoxP3^+^CD25^+^) in PBMC.

To explore whether the association between IPV and CD4^+^ T-cell activation may be due to deficient Treg activity, thawed PBMC were stained with anti-CD3, anti-CD4, anti-CD25, and intracellularly with anti-Foxp3, and acquired by flow cytometry. No association between past-year or lifetime IPV and frequency of Tregs (Foxp3^+^CD25^+^) was noted (Figure 4A, 4B, 5A, 5B). Next, to evaluate whether IPV was associated with changes in the phenotype of Tregs, anti-CD45RA and anti-HLA-DR were used to differentiate naïve (CD45RA^+^/HLA-DR^-^) from terminal effector (CD45RA^-^/HLA-DR^+^) Tregs. Higher scores of both past-year and lifetime IPV were associated with a shift in Treg phenotype from naïve (r = -0.327, *P* = 0.004 and r = -0.337, *P* = 0.003, respectively) to terminal effector (r = 0.282, *P*=0.014 and 0.225, *P* = 0.052, respectively) (Figure 4C, 4D, 5C, 5D). Higher frequencies of terminal effector Tregs were not significantly associated with CD4^+^ T-cell activation (r = -0.159, *P* = 0.174), but were negatively associated with CD8^+^ T-cell activation (r = -0.268, *P* = 0.020).

### Understanding the IPV-CD4 Activation Pathway

To evaluate whether the link between IPV and CD4 activation was confounded by other socio-demographic and environmental factors (Figure 1), the association between these covariates and CD4 activation was assessed (Table 1). Multivariable regression was used to explore confounding of the IPV-CD4 relationship. Race, the only covariate significantly associated with CD4 activation, was placed in the model. The association between lifetime IPV experience and CD4^+^ T-cell activation remained significant (β = 0.313, *P* = 0.012) while the association between past-year IPV and CD4^+^ T-cell activation was not (β = 0.197, *P* = 0.123).

Lastly, we sought to explore potential moderators of the IPV-CD4 activation pathway. Of all moderators tested (depression, PTSD, cortisol, cocaine, NSAID, and alcohol use), none were found to moderate the past-year IPV-CD4 activation nor lifetime IPV-CD4 activation pathways. Similar moderation analysis was conducted for different IPV subtypes. Significant interaction of the past-year physical IPV-immune activation pathway by depression (r_dep_ = 0.163, r_nondep_ =0 .486, *P* = 0.042) and alcohol use (r_alc_ = -0.194, r_nonalc_ = 0.323, *P* = 0.054) was noted, as was interaction of the lifetime physical IPV-immune activation pathway by alcohol use (r_alc_ = -0.144, r_nonalc_ = 0.451, *P* = 0.029). There were no other statistically significant moderators of the IPV subtype-immune activation pathways. The occurrence of STIs and statin use were infrequent (i.e. 1/75 had an active genital ulcer and positive HSV culture; 1/75 reported statin use), therefore evaluation of interaction by STIs and statins was not pursued.

## DISCUSSION

The increased HIV risk incurred by IPV survivors has been explained only in part through behavioral pathways. Through this pilot study of HIV-negative high-risk women, we present preliminary evidence for one biological pathway: IPV experience is associated with increased CD4^+^ T-cell activation. It has been well-established that individuals with increased frequencies of CD4^+^ T-cell activation have increased susceptibility to HIV infection [33–37], as activated CD4^+^ T cells allow for efficient viral entry, integration, replication, and release [38, 39, 48]. Although we are the first to report the link between IPV experience and CD4^+^ T-cell activation, other stresses have been linked to increased cellular activation. For example, recently released prisoners of war [49], women who suffer from PTSD from prior child abuse [50, 51], children who suffer abuse [52], women with depression [53], and individuals exposed to standardized laboratory stressors [54] have higher levels of CD4^+^ T-cell activation compared to unstressed controls.

We next sought to further characterize the IPV-CD4^+^ T-cell activation pathway. First, recognizing that memory CD4^+^ T-cells have a lower threshold for activation [55, 56], we phenotyped the CD4^+^ T-cells and found that experience of IPV was associated with increased frequency of effector memory (CD45RA^-^CD27^-^) CD4^+^ T-cells. Several studies have demonstrated that memory CD4^+^ T-cells bind HIV more effectively and are more susceptible to HIV infection than naïve CD4^+^ T-cells [57–60], thus possibly explaining how IPV experience may increase HIV risk. Then, recognizing IPV-associated stress is likely part of an “environment of stress” in this population, we attempted to measure each of the co-existent environmental stresses to evaluate for confounding (i.e. homelessness, low socio-economic status, discrimination, substance abuse, race, experience of child abuse, relationship status, and sexual orientation). In bivariate analysis, only race was associated with CD4^+^ T-cell activation and when entered into the model, lifetime IPV (but not past-year IPV, likely due to insufficient power), remained significantly associated with CD4^+^ T-cell activation. Lastly, all measured forms of IPV (i.e. physical, sexual, and psychological abuse) were associated with CD4^+^ T-cell activation, and after controlling for sexual abuse, the association between both physical and psychological abuse and CD4^+^ T-cell activation remained significant. This finding supports the hypothesis that the biological effects of IPV on HIV susceptibility go beyond the direct mucosal-level trauma resulting from forced sexual intercourse. The moderation of the IPV-CD4 activation pathways by alcohol use and depression, and not by other measured covariates, is difficult to interpret as the moderation analyses were underpowered; however, the results suggest that heavy alcohol use and/or depression “buffer” or “mask” the effects of IPV on CD4^+^ T-cell activation.

Because of recent studies linking HIV susceptibility to low frequencies of circulating Tregs [36, 40, 41] and the regulation of cellular activation by Tregs, we next aimed to evaluate whether IPV experience was associated with lower frequencies of Tregs but found no such correlation. The recent recognition of Tregs as a heterogeneous population of various subsets and diversity of function [61–63] led us to evaluate whether IPV was associated with a shift in Treg phenotype. We found that women who reported higher IPV exposure had relatively higher frequencies of terminally-differentiated effector (CD45RA^-^/HLA-DR^+^) Tregs, and that this terminal differentiation was negatively associated with CD8^+^ T-cell activation. The implications of these findings in terms of HIV susceptibility are unknown, but in HIV-infected populations, frequency of effector Tregs has been inversely correlated with HIV-specific CD8^+^ T-cell responses (i.e. activation and IFN-γ production) [64]. Furthermore, while terminally differentiated Tregs exert stronger suppression of T-cell responses [61, 62], they also die more rapidly and proliferate less compared with naïve Tregs [63]. Taken together, these studies lead us to speculate that IPV-induced terminal effector differentiation of Tregs may result in a more ‘exhausted’ Treg compartment with a less effective response upon HIV exposure. Alternatively, the increased terminal differentiation of Tregs may simply be a response to long-term IPV-associated CD4^+^ T-cell activation.

This pilot study possesses limitations common to many exploratory investigations. First, the cross-sectional design limits capacity to draw causal inferences. Although immune activation itself would be an unlikely determinant of IPV, other conditions with which CD4^+^ T-cell activation is associated may be determinants of IPV. We tried to minimize this possibility by measuring and accounting for other clinical and socio-behavioral variables affecting activation in the analysis. Second, our small sample size may have limited statistical power to detect confounding and moderation in the multivariable model. Third, substance abuse and alcohol use were assessed by self-report to single questions, and therefore may have been inadequately measured. Future large-scale studies should consider more effective means of measuring these potential confounders (i.e. validated scales and drug screening), and also consider assessment of confounding by other covariates not possible in this pilot study (i.e. CMV viremia, tobacco and methamphetamine use). Fourth, the study was conducted with a select sample (i.e. non-pregnant women without auto-immune disease or immunosuppression), therefore the generalizability of these findings to other HIV high-risk women is unknown. Lastly, our pilot study was limited to assessing whether IPV was associated with systemic CD4^+^ T-cell activation and Treg frequency and phenotype. Future studies should examine whether the increased activation experienced by IPV survivors translates into mucosal level cellular changes and increased *in vitro* HIV susceptibility, and whether the IPV-associated shift from naïve to terminal effector Tregs is associated with diminished functionality.

In conclusion, our findings suggest that the increased HIV susceptibility incurred by survivors of IPV may be in part due to increased immune activation. Although requiring validation on a larger scale and in other populations, our study opens a new field of investigation within HIV prevention research.
